# The composition of microbial communities in inflammatory periodontal diseases in young adults Tatars

**DOI:** 10.3934/microbiol.2021005

**Published:** 2021-01-27

**Authors:** Maya Kharitonova, Peter Vankov, Airat Abdrakhmanov, Elena Mamaeva, Galina Yakovleva, Olga Ilinskaya

**Affiliations:** 1 Institute of Fundamental Medicine and Biology, Kazan Federal University, Kremlevskaya Str. 18, Kazan 420008, Russia; 2 Department of Pediatric Dentistry, Kazan State Medical University, Butlerova Str. 49, Kazan 420012, Russia

**Keywords:** periodontitis, gingivitis, dental biofilm, young adult, oral microbial, α-diversity, TM7-3, Candidatus Saccharibacteria, *Rothia*, *Streptococcus*

## Abstract

Host susceptibility and environmental factors are important for the development of gingivitis and periodontitis, but bacterial biofilms attached to the teeth and gingival tissues play a crucial role. We have analyzed and compared the subgingival microbial communities between subjects with dental plaque biofilm-induced generalized chronic gingivitis (CG), localized initial (Stage I) periodontitis (IP) and healthy controls (HC) of young people aged 18–19 years permanently residing in the city of Kazan (Tatarstan, Russia). The results showed that the α-diversity in groups with CG and IP was higher than in the healthy group. In a course of periodontal disease, a decrease in the relative abundance of dominates genera Rothia and Streptococcus was observed along with increase of class TM7-3 (Candidatus Saccharibacteria phylum) representatives. Also, the increase of red complex representatives Porphyromonadeceae, Treponema and Tannerella was detected together with statistically significant increase of Filifactor, Parvimonas, Peptostreptococcaceae, Veillonellaceae, Tissierelaceae and Mogibacteriaceae. Analysis of our data suggests that transition from HC to IP may be accompanied by a decrease in microbial diversity and a reduction in the abundance of family Rs-045 (Candidatus Saccharibacteria phylum), Desulfovibrionaceae Corynebacterium, Campylobacter and Selenomonas in young adults Kazan Tatars.

## 1. Introduction

The most common forms of inflammatory periodontal diseases are gingivitis and periodontitis [1]. Dental plaque biofilm-induced gingivitis is characterized by gingival inflammation, swollen and bleeding gums. In the absence of treatment, it may progress to periodontitis, which is characterized by the formation of pathogenic periodontal pocket, tissue destruction and bone resorption [2]. Inflammation of the periodontium can be induced by dysbiosis of the commensal oral microbiota, aggravated by disease-associated bacterial species [2]–[4]. Among various microbiomes of the human body, the oral microbiome is one of the most diverse. The Human Oral Microbiome Database (eHOMD) includes a total of 772 microbial species in 16 phyla, as follows: Actinobacteria, Bacteroidetes, Chlamydiae, Chlorobi, Chloroflexi, Cyanobacteria, Euryarchaeota, Firmicutes, Fusobacteria, Proteobacteria, SR1, Saccharibacteria (TM7), Spirochaetes, Synergistetes, WPS-2. Of all the species, 57% are officially named, 13% are unnamed cultivated taxa and 30% are as yet unnamed and uncultivated taxa [5]. Herewith, a separate mouth contains only about 100 species [6]. The richness of the microbial landscape of the oral cavity determines the polymicrobial etiology of gingivitis and periodontitis. [7]. Next-generation DNA sequencing methods make it possible to determine the structure of various microbial communities with high accuracy [8]. To date, the list of microorganisms associated with periodontal disease continues to be specified [9].

Thus, periodontal diseases are inflammatory and can be considered as infectious diseases. In addition to the bacterial infections, other factors make an important contribution to the development of gingivitis and periodontitis. An inappropriate inflammatory reaction may occur due to complicated cooperation of environmental factors (diet, smoking), territorial affiliation, host susceptibility and commensal microbiota [2],[10]. The onset and progression of periodontitis are exacerbated by systemic diseases such as diabetes, rheumatoid arthritis, Alzheimer's disease, cardiovascular disease [1],[10]–[13]. Since the species pattern of oral biofilms can vary even in healthy individuals, it is impossible to make a correct comparison of microbial profiles between periodontally diseased and periodontally healthy subjects. Unfortunately, sometimes the choice of groups for microbiological analysis is carried out without taking these data into account. As a result, the intragroup heterogeneity does not allow to establish the exact contribution of certain microorganisms to the disease.

The current study was designed to analyze the composition of the microbial community in three groups of young people (biofilm-induced generalized chronic gingivitis (CG), localized initial (Stage I) periodontitis (IP) and healthy controls (HC)). We focused on the worsening of the periodontal health status among non-smoking non-vegetarian young adults Tatars without systemic disease and overweight, residing in the city of Kazan (Tatarstan, Russia).

We assumed that comparing microbial communities of young adults with minimized intragroup heterogeneity and variability could clearly demonstrate the presence or absence of differences between periodontitis-associated, gingivitis-associated and health-associated microbiota. Therefore, the objective of our study was to determine some markers of bacterial dysbiosis leading to the transition from periodontal health to the gingivitis and from gingivitis to periodontitis in young adults Kazan Tatars.

## 2. Materials and method

### 2.1. Study population and sample collection

#### 2.1.1. Individuals

All studied participants (35% of men, 65% of women) were Tatars permanently residing in the city of Kazan (Tatarstan, Russia). Before being included in the study, each participant met with the physician (E.V.M.) for a medical visit to review his or her medical history, current medical issues, medications, and a food diary. All patients were registered with a parodontologist (A.K.A.). The patients had carried out a comprehensive survey of periodontal tissues to the definition of clinical indices and orthopantomography. Orthopantomograms were done with the help of OPG Panoramic X-Ray Machine (Path Image Instruments Co. Shil Bagan, Kolkata, India). All subjects were evaluated clinically and radiographically to assess the following periodontal measurements: number of teeth, probing pocket depth (PPD), clinical attachment level (CAL), bleeding on probing (BOP) and alveolar bone loss. Eleven healthy control subjects (n = 11), twelve patients with dental plaque biofilm-induced generalized chronic gingivitis (n = 12) and thirteen with generalized initial (Stage I) periodontitis (n = 13) participated in this study, among patients applied to the dental clinic ‘KamilDent’ (Kazan, Russia) in the 2014–2017 years.

##### 2.1.1.1. Inclusion criteria

Subjects were between 18 and 19 years old, were not registered with other medical institutions, had >24 teeth present, had a similar type of nutrition (nonvegetarian dietary pattern). Non-vegetarians were defined as those who eat red meat, poultry, fish, milk, and eggs more than once a week. All subjects were diagnosed according to criteria described by The American Academy of Periodontology [14]. Each site was classified as healthy (no BOP, CAL ≤ 1 mm, and PPD ≤ 3 mm), exhibiting gingivitis (BOP, CAL ≤ 1 mm, and PD ≤ 3 mm), initial periodontitis (BOP, CAL ≥ 1 mm and ≤ 2 mm, and PD ≤ 4 mm). Gingivitis case was defined using a bleeding on probing score (BOP %), assessed as the proportion of bleeding sites when stimulated by a standardized (dimensions and shape) periodontal probe with a controlled (∼0.25 N) force to the apical end of the sulcus at six sites (mesio-buccal, buccal, disto-buccal, mesio-lingual, lingual, disto-lingual) on all teeth present. A patient was diagnosed as a generalized gingivitis case according to a BOP score > 30%. All periodontitis patients had radiographic horizontal bone loss. Periodontitis was characterized as localized if < 30% teeth involved. Gingival health was defined as <10% bleeding sites with probing depths ≤3 mm.

##### 2.1.1.2. Exclusion criteria

Subjects with diabetes, HIV, pregnancy, smoking, <24 teeth, mucogingival and orthodontic pathology, vegetarians, antibiotic and antiseptics treatment within 3 months before the current study, or daily intake of medication and those who did not meet the ethnicity requirements were excluded from the study.

Demographic and clinical characteristics of study subjects are presented in Table 1.

**Table 1.** Demographic and clinical characteristics of the study subjects.

	HC (n = 11)	CG (n = 12)	IP (n = 13)
Age, mean years (range)	18.3 (18–19)	18.5 (18–19)	18.4 (18–19)
Male/female	4/7	4/8	5/8
Body mass index, mean ± SD	22.36 ± 2.24	21.98 ± 2.22	23.59 ± 2.50
Number of teeth, mean	28.9	28.4	28.5
Surfaces with plaque, mean ± SD %	12.4 ± 8.1	41.4 ± 10.3	44.1 ± 18.5
BOP, mean ± SD %	2.10 ± 1.08	32.0 ± 6.1	33.4 ± 10.5
CAL, mean ± SD %	0.23 ± 0.10	0.62 ± 0.29	1.9 ± 0.31
PPD, mean ± SD %	1.51 ± 0.17	2.12 ± 0.37	3.70 ± 0.65
Bone loss, mean ± SD %	0	0	14.5 ± 2,4

Abbreviations: BOP: percentage of teeth that displayed bleeding upon probing; CAL: clinical attachment loss; PPD: probing pocket depth; HC: healthy controls; CG: chronical gingivitis patients; IP: initial periodontitis patients.

#### 2.1.2. Ethics approval of research

The study was conducted in accordance with the approved instruction for experiments involving human subjects (Permission of the Ethical Committee of the Kazan State Medical University, Kazan, Russia IRB00009490 IORG0007903, September 15, 2020). Written informed consent was obtained from all patients studied.

#### 2.1.3. Samples

Selected sites were isolated using cotton rolls. Sampling was conducted after removal of supragingival plaque with a sterile scaler. Samples were obtained with sterile Gracey curettes. In individuals with healthy periodontium, as well as from patients with gingivitis, samples were obtained from the gingival groove. Plaque samples from periodontal pockets were collected with one single vertical stroke [15]. The samples were collected from the 5 sites per individual. The collected samples were then placed in 2 mL microcentrifuge tubes and frozen at −20 °C until further DNA isolation and comparative analysis of microbial communities.

### 2.2. DNA extraction and sequencing of amplicon libraries

Total DNA was extracted and purified from the selected samples using the QIAamp DNA Mini Kit (Qiagen, Germany) according to the manufacturer's instructions. The total amount of extracted and purified DNA was further measured using a Nanodrop ND-2000 spectrophotometer (Wilmington, USA). The resulting total DNA was stored in a freezer at −20 °C.

The fragments of bacterial 16S rRNA genes were amplified by the barcoded primers Bakt_341F (′5-CCT ACG GGN GGC WGC AG-3′) and Bakt_805R (′5-GAC TAC HVG GGT ATC TAA TCC-3′) using Phusion High-Fidelity DNA polymerase (Thermo Fisher Scientific, USA), in three replicates for each sample. The resulting amplicon for each sample was combined and purified using Agencourt AMPure XP beads (Beckman Coulter, USA). The amount of DNA was determined using the Quant-iT dsDNA HS Assay Kit. Sequencing was performed on a MiSeq sequencer (Illumina, USA).

### 2.3. Bioinformatics processing, data and statistical analyses

The resulting sequences were analyzed using QIIME, version 1.9.1. Paired reads were combined. 10.8% of the sequences were chimeric and low quality, and were removed. The remaining sequences were grouped into operational taxonomic units (OTU) at 97% similarity (at least five sequences for OTU). OTU were assigned by the open reference method. For the taxonomic classification of sequences, an RDP classifier was used. Observed OTU numbers and Shannon diversity indices calculated as the indicators for alpha diversity. The Kruskal-Wallis test was used to determine differences in relative abundance of phylotypes between groups. R version 3.4.1 was used. Significance was set at P < 0.05.

### 2.4. Nucleotide sequence accession number

The reads are available in the SRA database under BioProject accession number PRJNA494044.

## 3. Results

### 3.1. Microbial diversity

In the present study, the structures of microbial communities of 36 samples of periodontal microbiota were analyzed using sequencing of fragments of bacterial 16S rRNA genes (regions V3 and V4). After combining the pairwise reads, the average length of the resulting sequences was 460 bp. On average, there were 34,600 sequences per sample. Sequences were then taxonomically classified based on similarity to known sequences.

**Figure 1.**
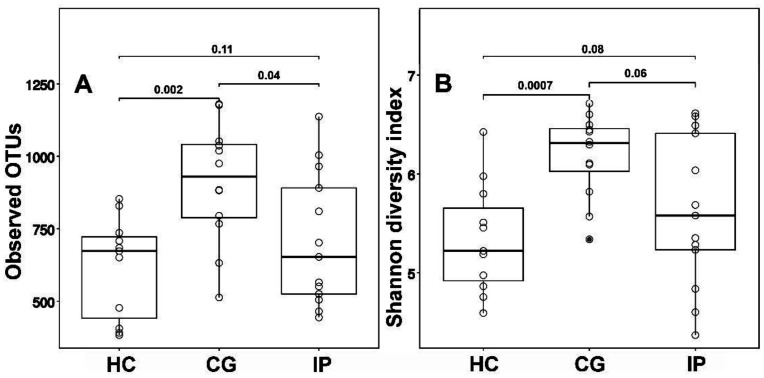
(A) Observed operated taxonomic units (OTUs) and (B) alpha diversities of subgingival plaques communities. CG - generalized chronic gingivitis, IP - localized initial periodontitis, HC-healthy controls. The Shannon index and number of OTUs are expressed with box plots (P value by 2-tailed Mann-Whitney-Wilcoxon test).

Bacterial diversity (determined by the Shannon diversity index) in groups of patients with dental plaque biofilm-induced generalized chronic gingivitis was significantly higher than in the healthy group. Median value of Shannon diversity index for the bacterial community of intact tissues was 5.1; of tissues affected by generalized chronic gingivitis was 6.2; of tissues affected by initial periodontitis was 5.4 (Figure 1). Microbial diversity was not significantly different between the IP and HC groups.

### 3.2. Subgingival microbial composition

Here, 183 phylotypes referring to 17 phyla were identified at the generic level. The median values of relative abundance of 47 most numerous phylotypes identified in three groups are presented in Table 2. Significant proportion of *Neisseria, Rothia, Fusobacterium, Prevotella, Veillonella* and *Actinomyces* was detected. Representatives of the genus *Streptococcus* predominated in most samples. The sequencing of bacterial 16S rRNA gene amplicons showed that in some samples from healthy tissue the *Streptococcus* count reached 50%.

**Figure 2.**
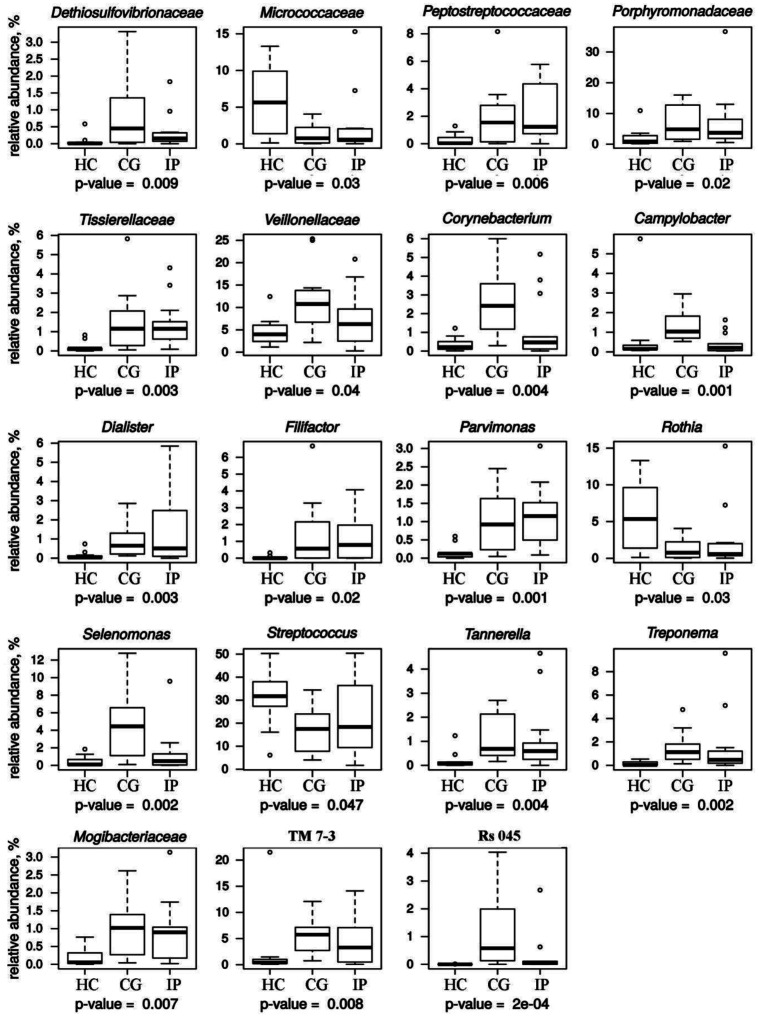
Relative abundance of bacterial groups (%), identified in three groups of juveniles with generalized chronic gingivitis (CG), localized initial periodontitis (IP) and healthy controls (HC). All abundances are based upon the proportional frequency of sequences that could be identified at the genus level and the family level (unclassified genus).

To determine all the detectable differences in the relative abundance of the phylotypes between the samples, the Kruskal-Wallis criterion was used. Figure 2 shows the distribution of reliably differing phylotypes whose median relative abundance exceeds 0.5% in at least one group. The statistical method used counted about 47 philotypes and the difference in these phylotypes between three groups was formally statistically significant, but a half of them presented in very small amounts. This fact did not allow us to consider these differences as important ones.

**Table 2.** List of relative abundance of bacterial groups (%), identified according to the results of the 16S rRNA sequencing. All abundances are based upon the proportional frequency of sequences that could be identified at the genus level, the family level (unclassified genus) and order-level (unclassified family).

Bacteria	healthy control	generalized chronic gingivitis	localized initial periodontitis
*Abiotrophia*	0.05 (0.00 to 0.55)	0.02 (0.00 to 3.40)	0.11 (0.00 to 7.24)
*Actinobacillus*	0.04 (0.00 to 1.91)	0.00 (0.00 to 0.33)	0.00 (0.00 to 7.59)
*Actinomyces*	2.46 (0.27 to 16.13)	1.50 (0.39 to 3.91)	1.49 (0.32 to 6.11)
*Aggregatibacter*	0.08 (0.01 to 3.18)	0.46 (0.00 to 3.54)	0.07 (0.00 to 3.18)
*Atopobium*	0.08 (0.00 to 2.60)	0.10 (0.00 to 0.99)	0.14 (0.01 to 1.63)
*Bacteroidales*	0.01 (0.00 to 0.05)	0.13 (0.01 to 1.95)	0.09 (0.00 to 1.41)
*Bulleidia*	0.07 (0.00 to 0.64)	0.14 (0.03 to 0.77)	0.20 (0.00 to 1.59)
*Campylobacter*	0.15 (0.08 to 5.76)	1.04 (0.53 to 2.96)	0.21 (0.05 to 1.63)
*Capnocytophaga*	0.85 (0.10 to 4.23)	1.85 (0.16 to 4.13)	2.31 (0.15 to 11.56)
*Comamonas*	0.10 (0.00 to 4.56)	0.01 (0.00 to 0.17)	0.04 (0.00 to 0.33)
*Corynebacterium*	0.21 (0.02 to 1.22)	2.42 (0.29 to 6.00)	0.47 (0.01 to 5.17)
*Desulfovibrionaceae*	0.01 (0.00 to 0.58)	0.44 (0.00 to 3.31)	0.16 (0.00 to 1.82)
*Dialister*	0.03 (0.01 to 0.74)	0.65 (0.12 to 2.85)	0.51 (0.00 to 5.58)
*Eikenella*	0.08 (0.01 to 0.64)	0.25 (0.04 to 2.65)	0.18 (0.00 to 1.48)
*Escherichia*	0.63 (0.04 to 26. 51)	0.15 (0.03 to 0. 95)	0.36 (0.01 to 2.78)
*Filifactor*	0.00 (0.00 to 0.32)	0.57 (0.00 to 6.68)	0.79 (0.00 to 4.07)
*Fusobacterium*	5.16 (0.39 to 14.97)	10.19 (3.36 to 21.73)	5.04 (1.19 to 30.09)
*Gemellaceae*	1.50 (0.05 to 6.38)	0.81 (0.30 to 5.90)	2.08 (0.08 to 14.66)
*Granulicatella*	3.46 (0.07 to 5.49)	0.63 (0.16 to 4.61)	1.38 (0.07 to 7.67)
*Haemophilus*	1.51 (0.00 to 14.65)	0.42 (0.07 to 10.16)	0.47 (0.01 to 6.52)
*Halomonas*	0.00 (0.00 to 3.60)	0.00 (0.00 to 0.00)	0.00 (0.00 to 0.29)
*Lachnospiraceae*	0.16 (0.01 to 6.48)	0.43 (0.00 to 2.91)	0.05 (0.00 to 1.84)
*Lautropia*	0.15 (0.01 to 2.33)	0.16 (0.02 to 1.28)	0.12 (0.00 to 3.20)
*Leptotrichia*	1.87 (0.14 to 16.97)	5.28 (0.01 to 19.83)	1.75 (0.03 to 10.59)
*Leptotrichiaceae*	0.00 (0.00 to 0.12)	0.00 (0.00 to 4.20)	0.00 (0.00 to 1.79)
*Megasphaera*	0.03 (0.00 to 0.75)	0.03 (0.00 to 2.39)	0.09 (0.00 to 1.19)
*Mogibacteriaceae*	0.06 (0.00 to 0.76)	1.02 (0.04 to 2.62)	0.90 (0.02 to 3.14)
*Neisseria*	8.50 (0.03 to 18.18)	0.65 (0.015 to 10.08)	1.84 (0.00 to 24.46)
*Oribacterium*	0.25 (0.00 to 2.14)	0.13 (0.00 to 0.51)	0.09 (0.00 to 1.45)
*Paludibacter*	0.05 (0.00 to 0.73)	0.36 (0.00 to 3.86)	0.19 (0.00 to 1.35)
*Parvimonas*	0.12 (0.00 to 0.60)	0.92 (0.05 to 2.45)	1.15 (0.09 to 3.07)
*Peptostreptococcus*	0.01 (0.00 to 0.65)	0.28 (0.00 to 1.31)	0.28 (0.00 to 5.47)
*Porphyromonas*	0.68 (0.02 to 9.74)	4.09 (0.29 to 12.36)	2.92 (0.53 to 32. 2)
*Prevotella*	3.27 (0.41 to 11.23)	5.75 (0.99 to 28.69)	4.74 (0.28 to 15.26)
*Rothia*	5.35 (0.13 to 13.30)	0.76 (0.03 to 4. 06)	0.57 (0.02 to 15.28)
*Rs-045*(Candidatus Saccharibacteria)	0.00 (0.00 to 0.01)	0.58 (0.00 to 4.04)	0.05 (0.00 to 2.67)
*Schwartzia*	0.00 (0.00 to 0.16)	0.74 (0.07 to 1.32)	0.19 (0.00 to 2.59)
*Selenomonas*	0.10 (0.00 to 1.85)	4.45 (0.10 to 12.78)	0.49 (0.00 to 9.59)
*Streptococcus*	31.73 (6.11 to 50.30)	17.51 (4.01 to 34.43)	18.37 (1.66 to 50.40)
*Tannerella*	0.07 (0.00 to 1.23)	0.69 (0.16 to 2.70)	0.59 (0.00 to 4.66)
*Tissierellaceae*	0.00 (0.00 to 0.33)	0.09 (0.00 to 3.82)	0.01 (0.00 to 1.22)
*TM7-3* (Candidatus Saccharibacteria)	0.41 (0.08 to 21.51)	5.75 (0.75 to 12.09)	3.28 (0.06 to 14.11)
*Treponema*	0.04 (0.00 to 0.54)	1.13 (0.13 to 4.76)	0.47 (0.00 to 9.57)
*Veillonella*	3.65 (0.36 to 10.19)	4.66 (0.47 to 11.89)	2.19 (0.00 to 7.64)
*Weeksellaceae*	0.18 (0.01 to 0.71)	0.09 (0.00 to 1.19)	0.18 (0.00 to 16.10)

The proportion of streptococci in healthy subgingival microbiota was about two times higher than in the CG and IP groups, the difference was statistically significant. Members of the genus *Rothia* as well as the entire *Micrococcaceae* family are also predominantly associated with periodontal health. The tendency of decrease in amount of *Neisseria* and *Actinomyces* was observed in disease. The difference between the three groups was not significant. However, it is worth noting that median values of their relative abundance in HC group were in 1.7 times (*Actinomyces*) and 4.6–13.1 times (*Neisseria*) higher than in the CG and IP groups.

We found the increase in the number of such bridgespecies as *Fusobacterium*, *Corynebacterium* and *Veillonella* in gingivitis-affected tissues. However, the proportion of these bacteria in IP group returned to a level comparable to that of the control group. Bridgespecies create the conditions necessary for colonization of plaque by pathogens such as *Porphyromonas gingivalis, Treponema denticola* and *Tannerella forsythia* [10],[16]–[18]. Genera *Treponema, Tannerella* and representatives of *Porphyromonadeceae* family, were significantly enriched in sites of subjects with CG and IP (Figure 2). Representatives of the genera *Filifactor, Parvimonas* and the *Mogibacteriaceae* family were found in greater proportion in CG and IP groups compared to periodontal health. Statistically significant results indicate the increase in proportion of *Filifactor*. Its median value did not exceed 1%, but in individual microbial community the abundance of *Filifactor* reached 6.68%. The same picture was observed for the family *Peptostreptococcaceae*, to which *Filifactor* belongs. The relative abundance of candidate class TM7-3 representatives significantly increased during the development of periodontal disease. TM7-3 belongs to Candidatus Saccharibacteria phylum (vernacular name: candidate division TM7). Saccharibacteria phylum is part of the superphylum Candidate Phyla Radiation. The data for the TM7-3 of the control group were exceeded in 8 (IP) and 14 (CG) times.

The average percentage of Rs-045 (belongs to the subgroup I025 of Candidatus Saccharibacteria phylum), *Desulfovibrionaceae* and members of the genera *Corynebacterium, Campylobacter* and *Selenomonas* was significantly higher in samples from gingivitis sites than in sites with initial periodontitis (Figure 2). The excess of relative abundance was 11.6 times (Rs-045), 2.8 times (*Desulfovibrionaceae*), 5.14 times (*Corynebacterium*), 5.0 times (*Campylobacter*) and 9.1 times (*Selenomonas*).

## 4. Discussion

Comparative analysis of the subgingival microbiome is critical for understanding the role of subgingival microbial communities in onset and progression of gingivitis and periodontitis, which are the most common forms of inflammatory periodontal disease. In the present study, we used 16S rRNA sequencing to determine the composition and structure of the subgingival microbiomes in young adults Tatars with gingivitis, initial periodontitis and healthy controls. The criteria for choosing age group (from 18 to 19 years) was, on the one hand, the completion of the formation of permanent teeth and the decrease in the influence of sympathetic innervation on the growth of the jaws (18 years), on the other, the end of the puberty period (19 years).

As in previous studies [19],[20] CG group was characterized by a statistically significant higher α-diversity compared to healthy group. At the same time, microbial diversity was not significantly different between the IP and HC groups. Similarly, no significant difference in species richness (number of OTUs) was observed between the IP and control HC groups. Kirst et al. also observed no significant difference in microbial diversity between healthy and periodontitis sites, although species richness was slightly higher in diseased sites [21]. Other previous studies reported greater microbial diversity and species richness in sites with periodontitis compared to healthy controls [7],[22]. In our study, the α-diversity in the IP group varied over a wide range due to the variety of individual microbial pattern [7],[10]. There is considerable inter-individual variability in the relative abundance of phyla. Indeed, the development of slowly or moderately progressing periodontitis is the result of a polymicrobial infection with variable microbial patterns. In opposite, the onset of rapidly progressing periodontitis is usually not commensurate with the amount of microbial deposits and a high diversity of microbial communities [10],[23].

Microbial communities of the healthy individuals are in balance with host immune response and contain mainly gram-positive facultative anaerobic *Streptococcus, Actinomyces, Rothia*. In this case, there are no signs of inflammation in the periodontium [11],[21]. Our data is in agreement with studies compared the healthy volunteers to the gingivitis and periodontitis patients [2],[19],[21]. The major proportion of the bacterial community from a healthy periodontium is commensal oral streptococci (*S. sanguis, S. mitis, S. oralis*), while highly acidogenic and aciduric *Streptococcus mutans* is a pathogen [21],[24]. If removed regularly, the dental biofilm mainly comprises oral streptococci relating to non-pathogenic resident microbiota [25]. In accordance with the study by Carrouel et al. (2016) and Bourgeois et al. (2017) the interdental biofilm of young periodontally healthy subjects contains bacteria that are able to induce periodontitis and caries. Our study also reveals that even dangerous periodontal pathogens such as the representatives of red complex can be found in samples from young periodontally healthy subjects [26],[27].

It is known that possible pathogenic bacteria are not able to colonize oral surfaces in the presence of commensal bacterial community due to the so-called ‘phenomenon of colonization resistance’ [2],[25]. After earliest colonizers, the so-called bridgespecies coaggregate late colonizers, which contribute to the development of pathologies. So, in a study that used spectral imaging FISH was shown that the Corynebacterium filament is encased in a corncob shell containing in particular *Porphyromonas* and frequently abundant *Haemophilus/Aggregatibacter* [16]. In the CG group such bridgespecies as *Fusobacterium, Corynebacterium* and *Veillonella* were more abundant than in HC and IP groups. Declination of the proportion of the bridgespecies in IP group was surprising, especially for *Fusobacterium*. According to Griffen et al., genera *Veillonella* and *Corynebacterium* did not show significant mean differences between healthy controls and deep pockets of patients with periodontitis [7]. Previous studies observed that *Fusobacterium* was one of the most abundant genus in the subgingival microbiota of periodontally healthy persons [28]. However, *Fusobacterium* was more abundant in subjects with periodontitis [7]. Although it has been recognized that *F. nucleatum* has periodontopathogenic properties [29], results of Camelo-Castillo et al. raise doubts on its role. A negative relationship of Fusobacterium with such clinical parameters, as pocket depth has been shown [28].

The most virulent or putative bacteria contributing to the emergence of adult periodontal disease are representatives of the red complex, which includes *Porphyromonas gingivalis*, *Treponema denticola* and *Tannerella forsythia* [25]. According to this data, we found the statistically significant increase of *Porphyromonadeceae*, *Treponema* and *Tannerella* in the group of city juveniles with CG and IP (Figure 2). Other bacterial species such as *Prevotella intermedia, Aggregatibacter actinomycetemcomitans, Fusobacterium nucleatum, Selenomonas noxia*, and *Eubacterium nodatum* are associated with periodontitis. Also, *Selenomonas, Synergistes, Desulfobulbus, Eikenella corrodens, Peptostreptococcus micros, Campylobacter rectus*, TM7 and *Filifactor alocis* are identified as possible periodontopathogens [3],[5],[7],[17],[30]. Genera *Filifactor* and *Parvimonas*, families *Mogibacteriaceae* and *Porphyromonadaceae* were significantly enriched in sites of subjects with GG and IP. *Filifactor alocis* has unique potential virulence characteristics that collectively can lead to the disease process. *Filifactor* alocis express Microbial Surface Components Recognizing Adhesive Matrix Molecules that play an important role in Gram-positive bacterial virulence by mediating adherence to and colonization of host tissues as an early step toward clinically manifested infection. *F. alocis* able to induce proinflammatory cytokines and able to interact with other microbial species forming a polymicrobial synergistic relationship can enhance its invasive capacity and cause chronic inflammation [3]. The genera *Peptostreptococcus* and *Parvimonas*, identified in our study at an elevated level in the CG and IP groups (Figure 2), were reported to have increased relative abundance levels in patients with oral squamous cell carcinoma [31]. Although the individuals in our study were non-smokers, the finding of an association of *Mogibacteriaceae* with initial periodontitis was similar to that for smokers, whose subgingival microbiota was characterized by a significant increase in unclassified *Mogibacteriaceaea* genera [32]. Representatives of the *Mogibacteriaceae* family are among the subgingival phylotypes significantly enriched in subjects with gingivitis and periodontitis [21],[32],[33]. Although subgingival microbiota of adolescence with rapidly progressing periodontitis demonstrate high association with *Aggregatibacter actinomycetemcomitans* [10],[23],[30], our 16S rRNA analysis did not show significant differences in abundance of *Aggregatibacter* between HC, GG and IP groups. Interestingly, previous study of subgingival biofilms from patients with rapidly progressing periodontitis in a Brazilian population demonstrated no association between *A. actinomycetemcomitans* and disease [34].

Previous studies by Brinig et al. reported that percentage of TM7 (Candidatus Saccharibacteria) rDNA in mild-periodontitis samples was significantly higher than in both healthy and severe-periodontitis samples [35]. TM7 was more abundant in the subgingival metagenome of subjects with gingivitis than in healthy control group [35].We also found further evidence of involvement of bacteria of candidate bacterial phylum TM7(Candidatus Saccharibacteria) in oral pathological processes. Although in previous studies [35] there was no correlation between the relative abundance of TM7 rDNA and age, we suggest an important role of TM7-3 in onset of young adults periodontal disease. Because abundance of TM7-3 in the studied age group (range: 18 to 19 years) was 6.1 times higher than in previous studies (age range: 23 to 74 years). Investigation of the influence of the salivary microbiome on the worsening of the periodontal health status among Japanese young adults showed higher proportion of TM7 with increase in PPD [36]. At the same time, the relative abundances of TM7 are comparable to the data obtained in our study. In studies by Rylev et al. an increase of TM7 phylum members (median 10.9%) was detected in patients with mucosal infections, however, there was no statistically significant difference in phylum proportions between the diseased and healthy subjects [37]. Across Saccharibacteria phylum have revealed host-associated members and first strain coisolated with its bacterial host (*Actinomyces odontolyticus*) from the human oral cavity was classified as epiparasite [38],[39]. Saccharibacteria are able to form dual-species biofilm communities with *Actinomyces oris, Fusobacterium nucleatum, Porphyromonas gingivalis, Prevotella intermedia, Parvimonas micra* or *Streptococcus gordonii* and promote biofilm formation via quorum sensing [40],[41]. TM7 bacteria possess genes necessary for production of Type IV pili and type II secretion apparatus, involved in pathogenicity and environmental adaptation [42],[43]. Therefore, accordingly to previous research and our results, ultrasmall TM7-3 bacteria are potentially associated with the early stages of young adults periodontal disease.

According to previous studies [2],[3],[5],[7],[11],[17],[21],[44], we also found an increased abundance of gram-negative anaerobic bacteria (*Fusobacterium, Veillonellaceae, Prevotella, Porphyromonadaceae, Treponema, Leptotrichia, Tannerella, Campylobacter, Capnocytophaga, Bacteroidales, Dethiosulfovibrionaceae*) in both groups with unhealthy periodontal tissues. A shift in the structure of the microbial community, characterized by an increase in the proportion of gram-negative anaerobic species, is considered as evidence of the development of periodontal disease [11]. Against the background of a significant decrease in nonpathogenic gram-positive bacteria, the appearance (*Filifactor*) and an increase (TM7-3, *Mogibacteriaceae*) of gram-positive bacteria, contributing to the pathogenetic structure of biofilm that cause periodontal inflammation, were significantly detected.

Representatives of the Rs-045 (Candidatus Saccharibacteria), *Desulfovibrionaceae* and also members of the genera *Corynebacterium, Campylobacter* and *Selenomonas* were found in a much higher proportion in samples of patients suffering from gingivitis than from initial periodontitis (Figure 2). Previous study of the cultivable subgingival microbiota in periodontal health, gingivitis and initial periodontitis by Tanner et. al 1998 [44] reported decrease in abundance of *Campylobacter gracilis* and *Selenomonas sputigena*. However, *Selenomonas noxia, Selenomonas flueggeii, Campylobacter rectus* were higher abundant in initial periodontitis group (age range: 20 to 60 years; % of subjects smoking: 17–83). Probably, the elimination of Rs-045 (Candidatus Saccharibacteria), *Desulfovibrionaceae Corynebacterium, Campylobacter* and *Selenomonas* with the course of periodontal disease could be an indicator of the transition of gingivitis to initial periodontitis exactly in young adults.

A number of studies have examined oral microbial communities of human hosts of different ethnic or environmental origin [36],[37],[45]–[47]. However, comparisons with our study are not always straightforward, since there are differences in the area of sampling (saliva, tonsils, supragingival and subgingival dental plaque) and the age of subjects. Premaraj et al. identified seven ethnic group-specific bacterial taxa among supragingival plaque of children between 6 and 11 years old in four ethnic groups (African American, Burmese, Caucasian, and Hispanic) and found that the microbial alpha diversity of supragingival microbiota significantly differed between ethnic groups [45]. Mason et al found that the subgingival microbial fingerprint could successfully discriminate between people four major ethnicities in the United States: non-Hispanic blacks, non-Hispanic whites, Chinese, and Latinos (over 18 years of age). Community diversity was higher in non-Hispanic whites group [46]. Kazan Tatars demonstrated higher bacterial diversity compared to all these ethnicities. Bacterial diversity in HC groups of the Kazan Tatars is similar with diversity of salivary microbiome of the German group and higher than that of the Alaskan group and the African group [47]. Representatives of genera *Capnocytophaga, Corynebacterium, Selenomonas, Prevotella* and *Aggregatibacter* found in Kazan Tatars were present in at least 80% of subgingival plaque of the subjects within non-Hispanic white's ethnicity [46].

We studied individuals without systemic diseases, which are a risk factor for periodontal disease and increase the severity of the disease. However, there is a bidirectional relationship between periodontal disease and systemic diseases [48]. Oral bacteria, bacterial products, and inflammatory molecules can invade the human body through the bloodstream or the digestive tract [49]. Periodontal pathogens are implicated in the most common noncommunicable diseases such as cardiovascular diseases, chronic obstructive pulmonary diseases, diabetes, rheumatoid arthritis, and cancer [49]. Oral pathogens may additionally serve as biomarkers for common noncommunicable diseases risk [50],[51]. *Porphyromonas gingivalis* and *A. actinomycetemcomitans* was associated with increased risk of pancreatic cancer [50]. *A. actinomycetemcomitans* has been recognized as a bacterial trigger for rheumatoid arthritis [52]. Serum antibodies to *Fusobacterium nucleatum* and *Prevotella intermedia* are a risk factor for Alzheimer's disease [53]. In the HC group, there were individuals with noticeably higher proportion of *Porphyromonas, Prevotella* and *Fusobacterium* than the median value. Representatives of these genera are associated with systemic diseases [51]. It can be assumed that some members of the HC group may develop systemic diseases. In addition to a biomarker, the oral microbiome can be both a tool and a target for treating diseases [51]. Dysbiosis of the oral ecosystem should be reversed to prevent systemic diseases. The systematic care management of periodontitis is indicated to reduce the risk of common noncommunicable diseases [49].

## 5. Conclusions

In conclusion, our study suggested that the worsening of the periodontal health status was associated with increased diversity and significant changes in microbial community structure and subgingival microbial composition in young adults with generalized gingivitis. The most indicative markers of the periodontal diseases development in city juveniles was a decrease in the relative abundance of dominate genera *Rothia (Micrococcaceae)* by about an order of magnitude, as well as twofold loss in the number of Streptococcus, along with increase of class TM7-3 representatives (Candidatus Saccharibacteria). Among the other groups, the increase of red complex representatives *Porphyromonadeceae, Treponema* and *Tannerella* was detected together with statistically significant increase of *Filifactor, Parvimonas, Peptostreptococcaceae, Veillonellaceae, Tissierelaceae* and *Mogibacteriaceae*. However, periodontitis initiation in young adults was characterized by a decrease in diversity and generally not associated with a further significant increase in abundance of disease-associated bacteria. At the same time, a decrease in the proportion of *Corynebacterium, Campylobacter, Selenomonas*, Rs-045 (Candidatus Saccharibacteria) and *Desulfovibrionaceae* may be an indicator of the transition of general gingivitis to initial periodontitis in non-smoking young adults.
